# Drug distribution and efficacy of the DprE1 inhibitor BTZ-043 in the C3HeB/FeJ mouse tuberculosis model

**DOI:** 10.1128/aac.00597-23

**Published:** 2023-10-04

**Authors:** Michelle E. Ramey, Firat Kaya, Allison A. Bauman, Lisa M. Massoudi, Jansy P. Sarathy, Matthew D. Zimmerman, Dashick W. L. Scott, Alyx M. Job, Jake A. Miller-Dawson, Brendan K. Podell, Michael A. Lyons, Véronique Dartois, Anne J. Lenaerts, Gregory T. Robertson

**Affiliations:** 1 Mycobacteria Research Laboratories, Department of Microbiology, Immunology and Pathology, Colorado State University, Fort Collins, Colorado, USA; 2 Center for Discovery and Innovation, Hackensack Meridian School of Medicine, Nutley, New Jersey, USA; Houston Methodist Academic Institute, Houston, Texas, USA

**Keywords:** tuberculosis, murine models, DprE1 inhibitor, BTZ-043, C3HeB/FeJ

## Abstract

BTZ-043, a suicide inhibitor of the *Mycobacterium tuberculosis* cell wall synthesis decaprenylphosphoryl-beta-D-ribose 2′ epimerase, is under clinical development as a potential new anti-tuberculosis agent. BTZ-043 is potent and bactericidal *in vitro* but has limited activity against non-growing bacilli in rabbit caseum. To better understand its behavior *in vivo*, BTZ-043 was evaluated for efficacy and spatial drug distribution as a single agent in the C3HeB/FeJ mouse model presenting with caseous necrotic pulmonary lesions upon *Mycobacterium tuberculosis* infection. BTZ-043 promoted significant reductions in lung and spleen bacterial burdens in the C3HeB/FeJ mouse model after 2 months of therapy. BTZ-043 penetrates cellular and necrotic lesions and was retained at levels above the serum-shifted minimal inhibitory concentration in caseum. The calculated rate of kill was found to be highest and dose-dependent during the second month of treatment. BTZ-043 treatment was associated with improved histology scores of pulmonary lesions, especially compared to control mice, which experienced advanced fulminant neutrophilic alveolitis in the absence of treatment. These positive treatment responses to BTZ-043 monotherapy in a mouse model of advanced pulmonary disease can be attributed to favorable distribution in tissues and lesions, retention in the caseum, and its high potency and bactericidal nature at drug concentrations achieved in necrotic lesions.

## INTRODUCTION

Despite the introduction of the current front-line drug regimen more than 40 years ago, tuberculosis (TB), caused by the bacterium *Mycobacterium tuberculosis* (*Mtb*), remains an urgent worldwide health problem (1, 2). In 2020 alone, more than 10 million new TB cases were documented, and there were roughly 1.5 million TB-associated deaths. Additionally, treatment and control of TB were adversely impacted by severe acute respiratory syndrome coronavirus 2 (SARS-CoV-2)-related disruptions in essential patient services (3). TB remains a potentially curable disease, but current standard regimens range in duration from 6 months for drug-susceptible TB infections to years for drug-resistant forms of the disease, and second-line therapies are associated with potential toxicities (4, 5).

Highlighting extensive efforts to develop new, more effective therapies, the TB drug development pipeline (www.newtbdrugs.org) includes multiple promising new anti-TB therapeutics at different stages of development, including three drugs (i.e., bedaquiline, delamanid, and pretomanid) that received regulatory approval for the treatment of drug-resistant TB (6). The field also witnessed the results of a landmark clinical trial conducted by the Tuberculosis Trials Consortium Study 31/AIDS Clinical Trials Group A5349 (Clinical Trials.gov Identifier: NCT02410772), which demonstrated the non-inferiority of a 4-month four-drug regimen by replacing two drugs in the 6-month standard of care with the existing anti-TB agents, rifapentine and moxifloxacin (7). Additionally, a 6-month regimen consisting of two drugs with novel modes of action (i.e., bedaquiline and pretomanid) and a third repurposed drug (i.e., linezolid) was approved in 2019 for the treatment of multidrug-resistant and extensively drug-resistant forms of the disease (8). Yet, despite these encouraging results, additional new drugs with a novel mechanism of action are still urgently needed to further shorten treatment and improve treatment safety profiles for all TB patient populations (9).

The decaprenylphosphoryl-beta-D-ribose 2′ epimerase (DprE1) is a mycobacterial enzyme that plays an essential role in cell wall biosynthesis and is a novel drug target in *Mtb* (10). DprE1 is a flavoprotein that works with decaprenylphosphoryl 2-keto-ribose reductase to catalyze the conversion of decaprenylphosphoryl-β-D-ribose to decaprenylphosphoryl-β-D-arabinose, the precursor of arabinans for cell wall biosynthesis for mycobacteria and Corynebacteriaceae species (11, 12). DprE1 is targeted by four novel drug candidates currently in clinical trials, including the benzothiazines (BTZs) BTZ-043 and PBTZ169, the azaindole TBA-7371, and the carbostyril drug OPC-167832 (10, 13 – 15). BTZ-043 and PBTZ169 are covalent suicide inhibitors of the enzyme, whereas OPC-167832 and TBA-7371 form non-covalent interactions as they lack a nitro substituent required for covalent interaction with DprE1 (10, 16 – 18). All four drugs exert potent bactericidal activity in mice (14, 15, 19, 20). More recently, PBTZ169, OPC-167832, and TBA-7371 were profiled head-to-head in the C3HeB/FeJ TB mouse infection model (21), which presents with a heterogeneity of pulmonary TB lesion types more reflective of human disease (22 – 32). That study confirmed the significant efficacy after 8 weeks of treatment (5 of 7 days per week) in C3HeB/FeJ mice, with an average of 1.5 log_10_ lung CFU reduction compared to untreated controls for TBA-7371 at 100 and 200 mg/kg *bis in die* (BID, twice daily) and for PBTZ169 at 50 and 100 mg/kg *quaque die* (QD, once daily), whereas an average of 3 log_10_ lung CFU reduction for OPC-167832 was reached at 5 and 20 mg/kg QD (21). Quite strikingly, the BTZ compound PBTZ169 showed efficacy only during the second month of treatment, with the highest kill rates observed of the three DprE1 inhibitors tested (21). Whether this reflects the different pharmacokinetics of PBTZ169 or is instead a general attribute of covalent DprE1 inhibitors remains to be determined.

BTZ-043 is a second BTZ compound that is in late-stage clinical development by the University Hospital, University of Munich (ClinicalTrials.gov Identifier: NCT04044001). BTZ-043 exhibits nanomolar antimicrobial activity against *Mtb*, with similar overall potency against drug-resistant strains (33). It is bactericidal against replicating bacilli (10), with limited activity against non-growing streptomycin-dependent *Mtb* strain 18b following depletion of streptomycin (34). BTZ-043 is highly active against intracellular mycobacteria in macrophages with a deduced minimal inhibitory concentration (MIC) of <10 ng/mL and exhibits good efficacy at reducing lung and spleen burdens in a Balb/c chronic TB infection model with evidence of dose dependence between 37.5 and 300 mg/kg with 1 month of treatment (10). In a second study employing the same model, BTZ-043 was found to reduce lung burdens by ~0.6 to 1 log at the recommended dose of 50 mg/kg with 1 mo of treatment (15).

The present study sought to complete a comprehensive evaluation of BTZ-043 in the C3HeB/FeJ murine chronic TB infection model. First described for tuberculosis by Kramnik et al. (and referred to as the “Kramnik mouse model”) (35), the lung pathology of infected C3HeB/FeJ mice presents with three distinct lesion types: collagen-encapsulated caseous necrotic lesions (Type I lesions), fulminant neutrophilic alveolitis (Type II lesions), and cellular non-necrotizing lesions (Type III lesions), with only the latter lesion type being present in the BALB/c chronic TB infection model (24). These distinct pulmonary microenvironments are known to impact the *in vivo* activity of certain drugs (28, 36, 37). The impact on antibiotic effectiveness is influenced not only by the variable lesion architecture leading to drug- and lesion-specific pharmacokinetic properties (36, 38 – 40) but also by different lesion microenvironments leading to distinct bacterial populations within a given host (21, 41). To further understand the behavior of late-stage DprE1 inhibitors when given as monotherapy in a single mouse model with advanced pulmonary disease (21), BTZ-043 was profiled *in vitro* and in the C3HeB/FeJ murine tuberculosis model for efficacy and lesion-focused drug distribution. Although separate assays and animal infections were employed, these studies were designed to complement and extend earlier published studies with three other late-stage DprE1 inhibitors (21).

## RESULTS

### *In vitro* activity of the DprE1 inhibitor BTZ-043

**FIG 1:**
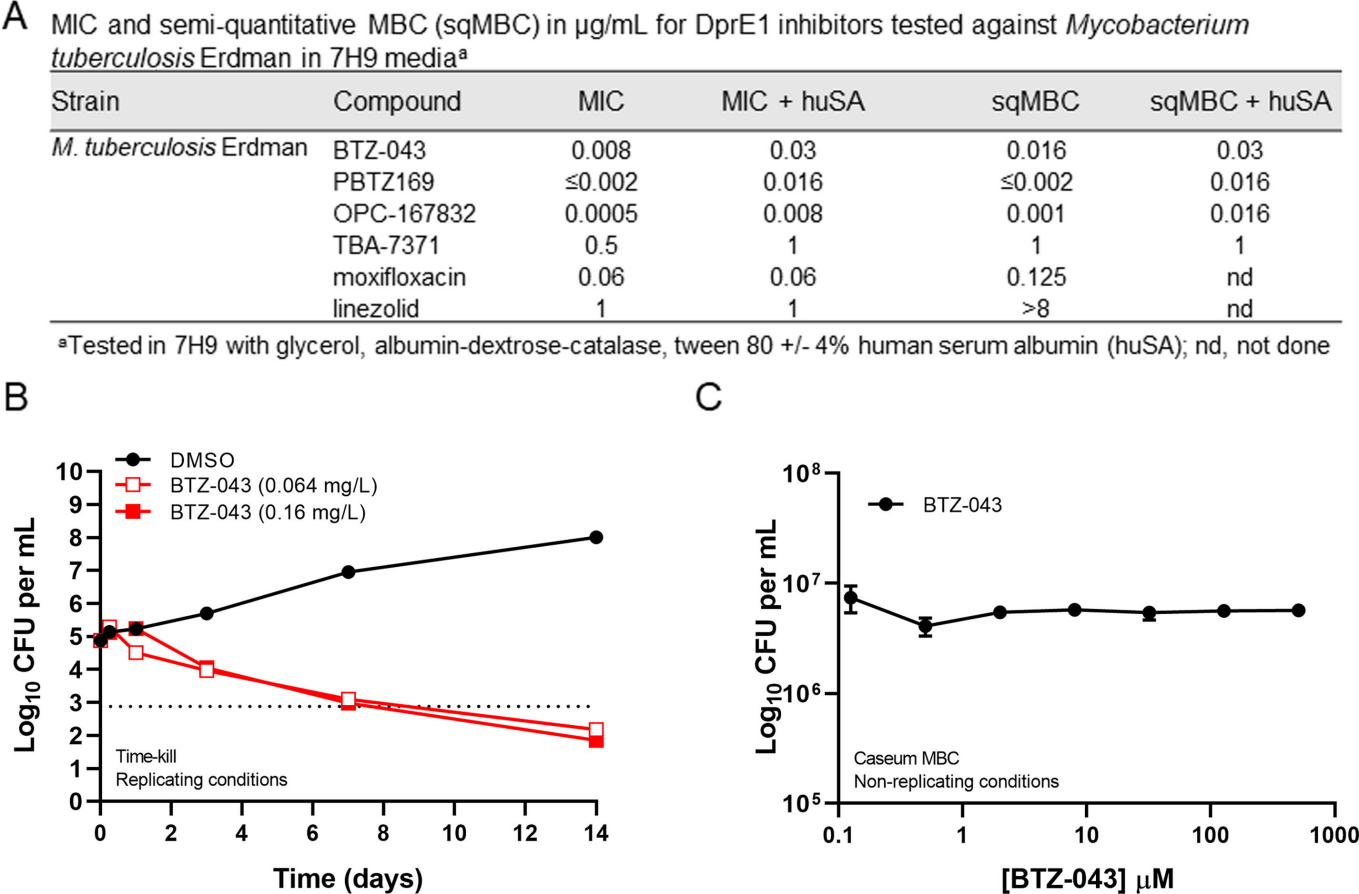
*In vitro* antimicrobial activity of BTZ-043 against actively replicating *Mtb* and non-replicating bacteria. (**A**) MIC and semi-quantitative MBC values in the presence and absence of 4% huSA. (**B**) *In vitro* kill kinetics at 8× and 20× the MIC of BTZ-043 compared to the DMSO vehicle negative control using *M. tuberculosis* Erdman under replicating conditions (representative of two independent assays). The dashed line indicates the 2-log kill, or 99% CFU reduction, of the starting inoculum. (**C**) Bactericidal activity of BTZ-043 using an *in vitro* assay with rabbit caseum. The data are expressed as the average log_10_ CFU per milliliter of homogenized caseum from three replicates. Standard deviations are indicated by error bars.

The *in vitro* activity of BTZ-043 was evaluated against actively replicating *Mtb* as well as against non-replicating bacteria in caseum. In a conventional broth microdilution assay with *Mtb* Erdman, the MIC value for BTZ-043 was 0.008 µg/mL (Fig. 1A). Relative to the other tested DprE1 inhibitors, BTZ-043 was less potent than OPC-167832 and PBTZ169 but appreciably more potent than TBA-7371. BTZ-043 showed a fourfold shift when assayed in the presence of physiologic levels of human serum albumin (huSA) found in whole blood (i.e., serum-shifted MIC), indicating ~75% protein binding by this assay (Fig. 1A). BTZ-043 was previously reported to show 99.4% plasma protein binding in a dialysis assay using human plasma (42), suggesting interaction of BTZ-043 with additional factors found in human plasma. All four tested DprE1 inhibitors exhibited cidality within twofold of their MIC following 10 d of incubation by a semi-quantitative MBC (sqMBC) assay measuring a 2-log kill or a 99% reduction in the inoculum (Fig. 1A). Moxifloxacin, a bactericidal fluoroquinolone, and linezolid, a bacteriostatic protein synthesis inhibitor, were included as positive and negative controls in the sqMBC assay, respectively (Fig. 1A). *In vitro* kill kinetics against *Mtb* Erdman under replicating conditions confirmed the cidality of BTZ-043 at 8× and 20× MIC (Fig. 1B). Results showed similar kill kinetics at both test concentrations as determined by CFU/mL over 14 days by serial dilution and culture on 7H11 agar plates with 0.4% activated charcoal to help counteract drug carryover artifacts (Fig. 1B). As is expected for cell wall synthesis inhibitors, BTZ-043 lacked appreciable activity when tested against non-replicating *Mtb* in an explanted rabbit caseum MBC assay (43) even at test concentrations of 512 µM (Fig. 1C). This was similarly reported previously for three other DprE1 inhibitors, namely, PBTZ169, TBA-7371, and OPC-167832 (21).

### Efficacy of BTZ-043 in the lungs of C3HeB/FeJ mice

The efficacy of BTZ-043 was evaluated in *Mtb* Erdman-infected C3HeB/FeJ mice after 4 and 8 weeks of daily drug treatment for 5 of 7 days per week (Monday-Friday) at 50, 100, and 200 mg/kg. Treatment was initiated 8 weeks post-aerosol infection to allow sufficient time for mice to develop pulmonary caseous necrotic lesions. The BTZ-043 dose range was centered on the reported individual minimal effective dose, resulting in a 1-log reduction in the bacterial load in the lungs versus untreated controls after treatment in a chronic *Mtb* murine infection model (15). As expected, the lungs of C3HeB/FeJ mice at the start of treatment presented with the typical spectrum of lesion types, with most mice showing the characteristic caseous necrotic lesions. The bacterial load in the lungs of the untreated, infected control C3HeB/FeJ mice showed a slight increase over time (6.80 log_10_ CFU ± 0.57 SEM at 8 weeks after aerosol, 7.88 log_10_ CFU ± 0.97 SEM at 12 weeks after aerosol, and 8.19 log_10_ CFU ± 0.64 SEM at 16 weeks after aerosol).

**FIG 2:**
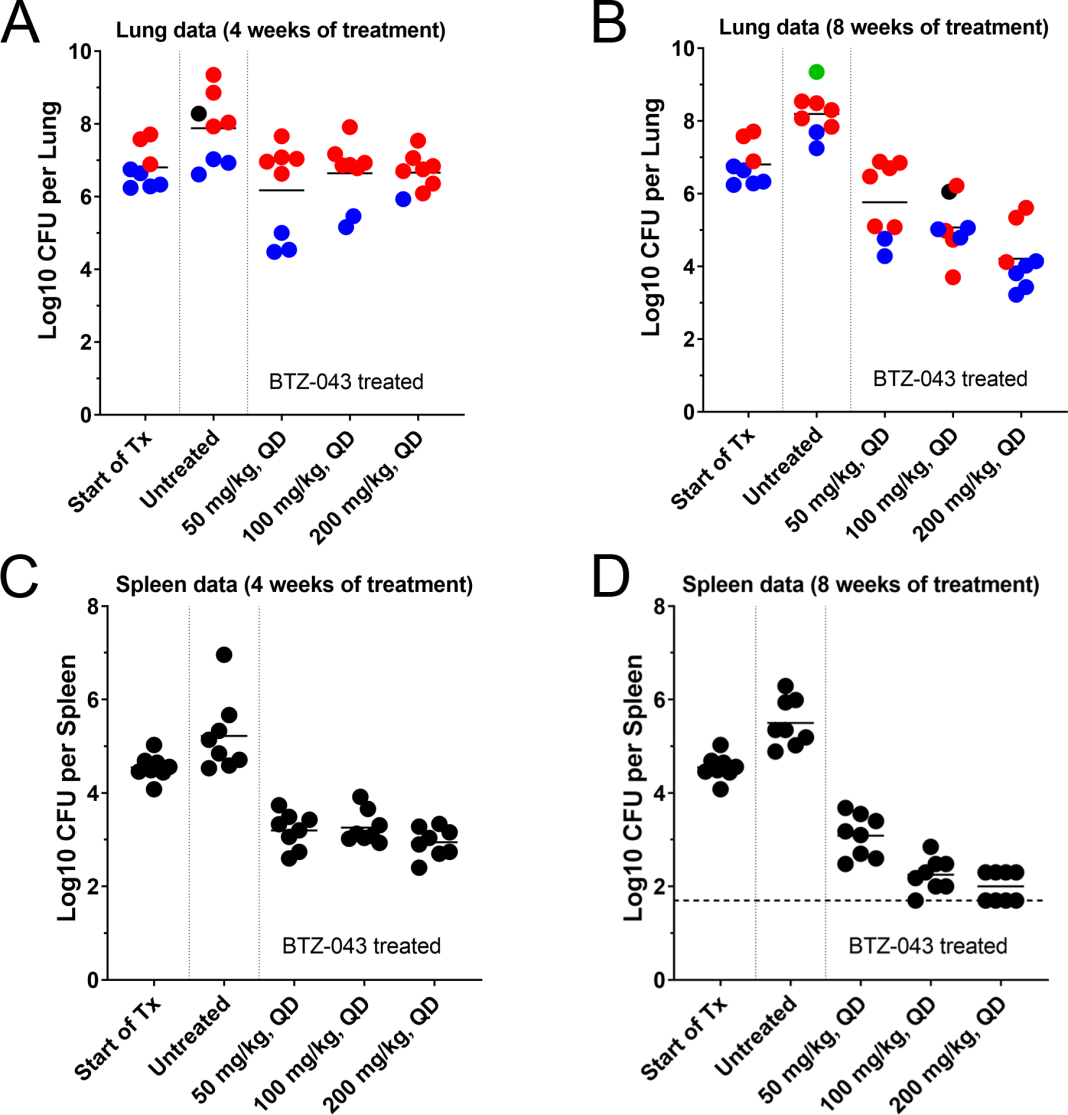
Efficacy of BTZ-043 in C3HeB/FeJ mice after 4 and 8 weeks of treatment. Eight weeks following a low-dose aerosol infection, mice were treated 5/7 days by once daily gavage (QD) at the doses indicated. Plots represent log_10_ CFU determinations in the lungs (**A and B**) and spleens (**C and D**) of individual C3HeB/FeJ mice after 4 (**A and C**) and 8 weeks (**B and D**) of treatment. Pre-treatment controls (start of treatment) represent untreated mice at the start of treatment (8 weeks post-aerosol infection). Lung pathology analysis based on blinded digital photographs taken at gross necropsy was used to classify mice as having pronounced Type I caseous necrotic lesions (red), lacking pronounced Type I lesions and presenting with only small Type I and/or Type III inflammatory lesions (blue), showing possible Type I lesions that were unclear in blinded photographs (black), or presenting with Type II fulminant neutrophilic alveolitis lesions (green). The horizontal dashed line depicts the lower limit of CFU detection in spleens (1.7 log_10_ CFU/spleen).

In lungs after 4 weeks of treatment, all BTZ-043 dose groups promoted statistically significant reductions in lung bacterial load (Dunnett’s, *P* = 0.002 to 0.03). There was no evidence of dose-proportional killing in the lungs with 4 weeks of treatment (Tukey’s, *P* = 0.64 to >0.99; Fig. 2A; Table S1). After 8 weeks of treatment, BTZ-043 groups showed a statistically significant reduction in lung bacterial load (Dunnett’s, *P* < 0.0001) (Fig. 2B; Table S1).

The calculated rate of kill (k, Δlog_10_ CFU/day) in lungs for mice treated with BTZ-043 was modest and lacked dose dependence during the first month of treatment (i.e., 0.022/day, 0.006/day, and 0.005/day for the low, intermediate, and high doses of BTZ-043, respectively), but showed to be robust and dose-dependent during the second month for mice treated with BTZ-043 (0.015/day, 0.056/day and 0.087/day for the low, intermediate, and high doses of BTZ-043, respectively; see Table S2.A). After 8 weeks of treatment, BTZ-043 at 200 mg/kg showed statistically higher reductions in bacterial lung load (i.e., ~3.98 log_10_ CFU reduction versus untreated and ~2.59 log_10_ CFU reduction from the start of treatment) than that observed at 50 mg/kg, the lowest dose of BTZ-043 tested (i.e., ~2.43 log_10_ CFU reduction versus untreated and ~1.04 log_10_ CFU reduction from the start of treatment) (Fig. 2B; Table S1).

### Caseous necrosis impacts the efficacy of DprE1 inhibitors in the lungs of C3HeB/FeJ mice

Treatment of C3HeB/FeJ mice with single agents is often associated with distinct treatment responses based on histopathology, whereby two distinct populations of mice emerge defined as “responsive” in mice with less severe pathology and “less responsive” in animals with severe pulmonary pathology, as described in previous studies (21, 26, 37). The most sterilizing compounds are hypothesized to show activity against bacteria present in both mouse populations. Therefore, studying changes in treatment response based on the presence of different lesion types can provide valuable additional information about the compound in development. Visual inspection of lungs was performed on blinded digital images taken from whole lungs at gross necropsy to distinguish mice with and without visible Type I (TI) lesions and determine if treatment response measured by CFU could be directly linked to the heterogeneous histopathology observed in the lungs of individual animals. The animals that presented with small TI lesions (not visible by eye at gross necropsy) as well as Type III (TIII) lesions were generally more responsive to BTZ-043 treatment (in blue, Fig. 2A and B). In contrast, animals with the most advanced necrotic lesions showed a slight trend toward an overall reduction in efficacy for BTZ-043, especially during the first month of treatment. Interestingly, several animals scored as having TI lesions by gross pathology were found to be responsive to BTZ-043 therapy during the second month (in red, Fig. 2B).

To determine whether BTZ-043 exhibited lesion-specific kill kinetics, kill rates were determined in mouse populations with predominantly large TI lesions as well as in mice lacking visible TI lesions and showing TIII pathology only. A higher rate of kill was observed during the first month of treatment for BTZ-043 in the 50 or 100 mg/kg dose groups with predominantly TIII lesions compared to those with T1 lesions, but the opposite trend was observed during the second month of treatment (Table S2B). Kill rates for the 200 mg/kg high-dose BTZ-043 group were similar in mice with TI and TIII lesions, with higher kill rates being observed during the second month of treatment (Table S2B).

### Efficacy of DprE1 inhibitors in the spleens of C3HeB/FeJ mice

In spleens, the bacterial load for the untreated, infected control C3HeB/FeJ mice showed a slight increase over time (4.55 log_10_ CFU ± 0.27 at 8 weeks after aerosol, 5.22 log_10_ CFU ± 0.80 at 12 weeks, and 5.50 log_10_ CFU ± 0.51 at 16 weeks). As previously described, the spleens in the C3HeB/FeJ mouse model show solely cellular lesions, as observed in the classical BALB/c chronic TB mouse infection model (35).

In spleens after 4 weeks of treatment, all dose groups of BTZ-043 promoted statistically significant reductions in bacterial load (Dunnet’s, *P* = 0.003 to 0.0001). There was no evidence of dose-proportional killing in spleens with 4 weeks of treatment (Tukey’s, *P* = 0.34 to 0.99; Fig. 2C). After 8 weeks of treatment, BTZ-043 promoted statistically significant reductions in spleen burdens (Dunnett’s, *P* < 0.0001), which were dose proportional as the dose increased from 50 to 100 mg/kg (Tukey’s, *P* = 0.01) and again from 50 to 200 mg/kg (Tukey’s, *P* = 0.005). The best overall efficacy was observed in the BTZ-043 high-dose group (200 mg/kg), where the CFU of 4 out of 8 mice was below the lower limit of detection (Fig. 2D). The difference between the intermediate (100 mg/kg) and high (200 mg/kg) dose groups was not statistically significant owing to several mice being reported at the lower limit of detection for the latter group (Tukey’s, *P* = 0.51).

In contrast to the lung data, kill rates for BTZ-043 in spleens showed more rapid killing during the first month of treatment and slower kill rates during month 2 of treatment (see Table S2C). This was especially evident for BTZ-043 at 50 mg/kg, where kill rates declined between month 1 and month 2 of treatment, whereas kill rates remained high for the 100 and 200 mg/kg dose groups. This suggests that a higher dose level may prove beneficial for BTZ-043 in spleens during the second month of treatment (see Table S2C).

### Effects of DprE1 inhibitors on the pulmonary histopathology of C3HeB/FeJ mice

**FIG 3:**
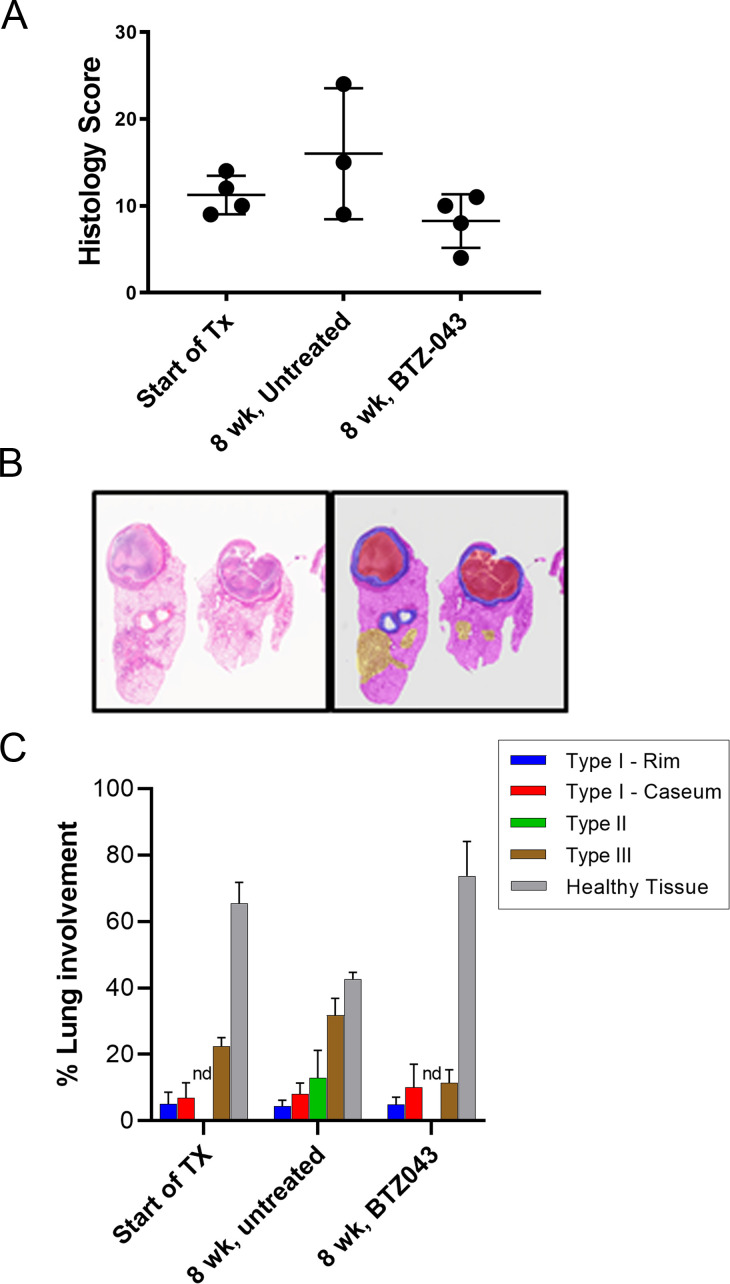
Histopathology analysis of pulmonary sections of C3HeB/FeJ mice at the start of treatment (Start of Tx) and in untreated controls (8 weeks, untreated) or after 8 weeks of treatment with BTZ-043 (at 200 mg/kg, QD). (**A**) Histopathology analysis on blinded samples by a board-certified pathologist using a semi-quantitative method based on histology scores (44). (**B**) Visual representation of the results generated by LIRA for digital images of pulmonary lesions of drug-treated C3HeB/FeJ mice. The various lesion classifications are displayed in a color overlay depicting Type I—rim (blue), Type I—caseum (red), Type II (green), Type III (brown), and healthy tissue (gray). A hematoxylin and eosin-stained image is included for reference (left panel). (**C**) Quantitative analysis plus the SEM using LIRA (22). nd, none detected.

Disease progression was monitored in untreated *Mtb* Erdman-infected C3HeB/FeJ mice and compared to mice receiving 2 months of treatment with BTZ-043 at 200 mg/kg during the same period. Qualitative histopathology analysis performed by a board-certified pathologist on blinded hematoxylin and eosin (H&E) stained tissue sections revealed that BTZ-043 was able to halt the progression of lung pathology from the start of treatment with improved overall histology scores compared to untreated mice at 8 weeks of treatment (Fig. 3A; Table S3). Similar results were obtained using digital scans of H&E-stained tissue sections and a new quantitative software called LIRA (“Lesion Image Recognition and Analysis”) (22), developed in our laboratory for digital image analysis of pulmonary pathology in *Mtb*-infected C3HeB/FeJ mice (Fig. 3B; Table S3). LIRA revealed a high incidence of Type III cellular lesions (22% of the total lung) and a lower incidence of Type I necrotic lesions (7% of the total lung) in *Mtb* Erdman-infected C3HeB/FeJ mice at 8 weeks (the start of treatment; Fig. 3C; Table S3A), consistent with the visual readout data. Progressive disease was evident in untreated mice 8 weeks later, where LIRA showed a small increase in Type III cellular lesions (32% of the total lung) and Type I necrotic lesions (8% of the total lung) and a marked increase in fulminant neutrophilic alveolitis Type II lesions (13% of the total lung). In contrast, in mice that received 8 weeks of BTZ-043 at 200 mg/kg, the incidence of Type III cellular lesions was lower than that observed at the start of treatment or in the untreated controls (11% of the total lung; Fig. 3C; Table S3C). BTZ-043 treatments also appeared to halt or prevent the progression of fulminant neutrophilic alveolitis Type II lesions (0% of the total lung), which represents a marked improvement compared to the untreated controls (13% of the total lung; Fig. 3C; Table S3B). There was a negligible difference in the appearance of Type I necrotic lesions between the BTZ-043-treated mice (10% of the total lung) and the untreated mice (8% of the total lung; Fig. 3C; Table S3B and S3C).

### Pharmacokinetics and tissue distribution of BTZ-043 in C3HeB/FeJ mice

**FIG 4:**
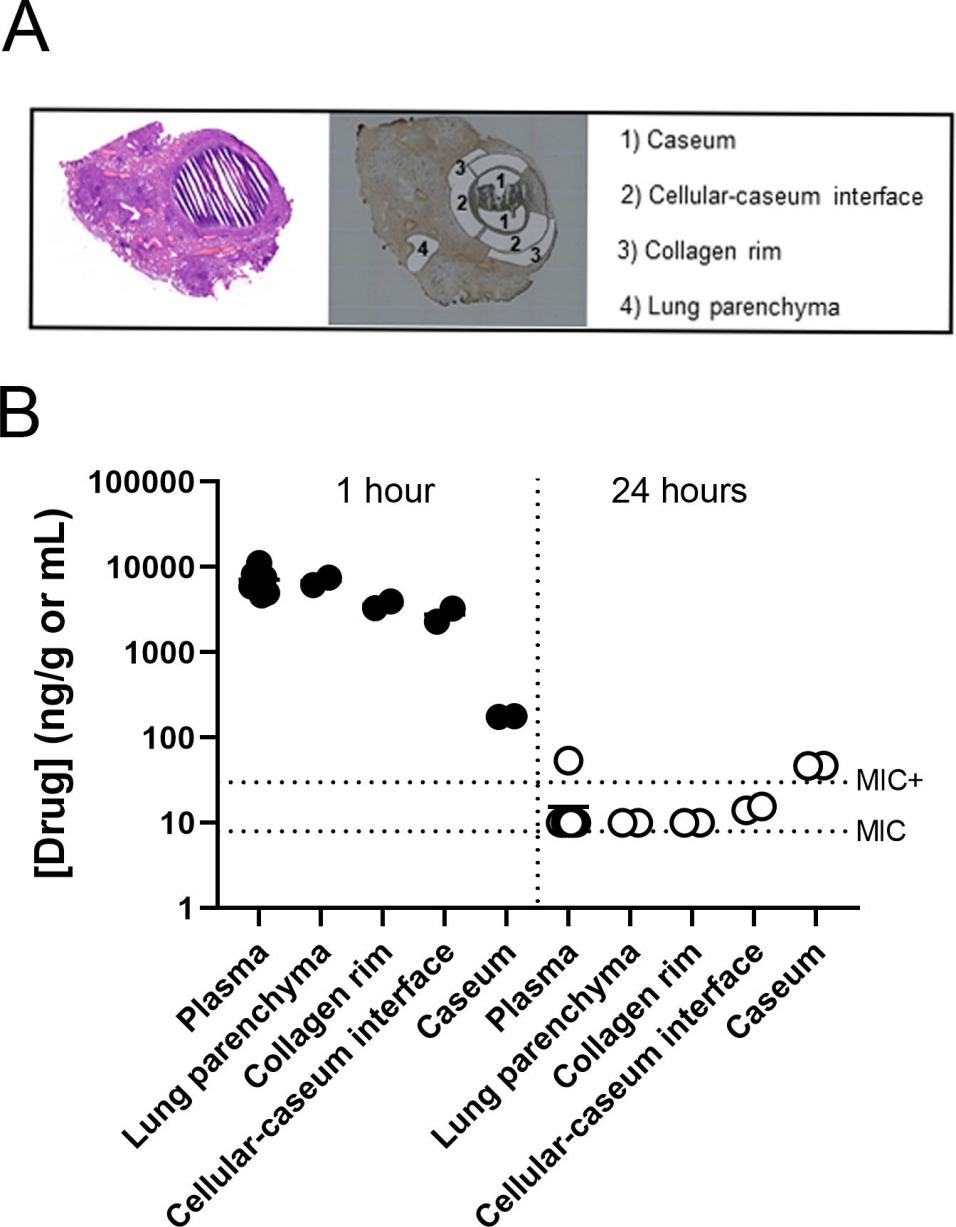
Quantitation of drug levels found in plasma and pulmonary lesions in *Mycobacterium tuberculosis*-infected C3HeB/FeJ mice after 2.5 weeks of treatment with BTZ-043 (100 mg/kg, QD). (**A**) Visual representation of areas collected by LCM for analysis. A hematoxylin and eosin-stained lung section image is included for reference (left panel), and areas sampled by LCM are shown (right panel). (**B**) Pharmacokinetic results presented employed the LCM-LC/MS-MS approach for quantification of the BTZ-043 in plasma and tissue samples, at plasma peak [1 h] or trough [24 h] time points. Dotted horizontal lines indicate the MIC (MIC) and MIC in the presence of 4% human serum albumin (MIC+) for the individual compounds. The detection limit of BTZ-043 was 10 ng/g by LCM-LC/MS-MS and 2 ng/mL in plasma.

The relationship between exposure and treatment response (pharmacokinetics-pharmcodynamics [PK-PD]) in lesions and how this correlates with efficacy was subsequently studied in dedicated groups of C3HeB/FeJ mice infected by low-dose aerosol with *Mtb* Erdman. Ten weeks post aerosol, mice received BTZ-043 5 out of 7 days per week at 100 mg/kg for 2.5 weeks to reach a steady state in tissues. Plasma samples and tissues were collected at 1 h (T_max_ relative to peak plasma concentration) and at 24 h (T_min_ or the end of the dosing interval). Tissue drug levels were quantified by gravity-assisted laser capture microdissection (LCM) in thin sections of specific lesion areas, followed by liquid chromatography coupled to mass spectrometry (LC/MS-MS). The benefit of the LCM approach is the ability to obtain absolute drug levels in defined areas of Type I lung lesions (24) such as the caseum (the core of the caseous necrotic lesion), foamy macrophage rim, necrotic neutrophil rim, and uninvolved lung parenchyma without cross-contamination. Four different areas were sampled by LCM within mature caseous necrotic pulmonary lesions: (i) acellular caseum, consisting of cellular debris with high numbers of extracellular bacteria shown previously to exhibit limited bacterial replication by rRNA synthesis ratio (RS ratio^®^) (21, 45); (ii) cellular-caseum interface, consisting of intact foamy macrophages and neutrophils with high numbers of intracellular, replicating bacteria (21, 45); (iii) the collagen rim; and (iv) uninvolved lung parenchyma (Fig. 4A). BTZ-043 showed a gradient of measured drug exposures at T1 (1 h), decreasing from high to low throughout the necrotic lesion: uninvolved lung parenchyma>collagen rim>cellular-caseum interface (cellular)>caseum (acellular and non-vascularized) (Fig. 4B). Drug levels were high and similar in plasma and whole lung and well above the MIC and the MIC in the presence of 4% huSA (MIC+) at C_max_. Appreciably lower levels of BTZ-043 were observed under these testing conditions at C_max_ in caseum (Fig. 4B). At trough, drug levels fell below the MIC+ in most tissue compartments, except for caseum, where measured drug levels remained above the MIC+ throughout the dosing interval (Fig. 4B). Similar results were obtained by standard tissue PK techniques using high-pressure LC/MS-MS of lung and lesion homogenates, which showed the highest drug levels at the trough in whole TI lesions and caseum, with drug levels in the other tissue samples at, or just below, the MIC+ (data not shown).

## DISCUSSION

New chemical entities capable of treating all disease forms of human TB are needed to improve treatment outcomes and shorten treatment duration, especially in patients with drug-resistant TB. Several chemical inhibitors of the Mtb DprE1 enzyme have emerged as novel drug candidates with excellent anti-Mtb properties *in vitro* and *in vivo* in conventional TB murine efficacy models. One such inhibitor, BTZ-043, a covalent suicide DprE1 inhibitor, was shown previously to exhibit potent antimicrobial activity *in vitro* and in conventional TB mouse efficacy models (10, 15). BTZ-043 has been extensively characterized following oral dosing in a variety of pre-clinical animal models (15, 46, 47). More recently, pharmacokinetic assessments of BTZ-043 exposures were also conducted in guinea pigs (48). In the current study, we profiled BTZ-043 for efficacy and spatial drug distribution using the murine C3HeB/FeJ chronic TB infection model. In the C3HeB/FeJ mouse model, the majority of bacteria are located in caseous necrotic Type I pulmonary lesions, with a high burden of intracellular bacteria in the foamy macrophage layer with neutrophils on the inside of the collagen rim (cellular-caseum interface) and an even higher proportion of extracellular bacteria in the caseum core (24). These lesional microenvironments influence antibiotic effectiveness in two ways. First, differences in vascularization, cellular composition, and variable architecture may impede drug partitioning and retention in lesions relative to levels found in healthy lung tissue (28, 36 – 38, 40, 49). Second, to be effective, the drugs must retain activity against the bacterial phenotypes that arise within these diverse tissue microenvironments, possibly owing to differences in pH, oxygen tension, and available carbon sources (28, 37, 41, 43, 50). This study was designed to complement and extend earlier published work that profiled three additional late-stage DprE1 inhibitors in the same model system (21).

*In vitro* assessment of BTZ-043 activity revealed excellent MIC potency, which was approximately fourfold less potent than the piperazine-containing BTZ derivative PBTZ-169, specifically designed to improve the pharmacological properties of the molecule (15). OPC-167832 showed the best overall activity by MIC, with lower *in vitro* potency observed for TBA-7371. All compounds proved bactericidal within twofold of their respective MIC values by the sqMBC assay. Serum shift MIC assays using physiologic levels of huSA revealed a 2-fold shift in MIC for TBA-7371, an 8-fold shift for both BTZ-043 and PBTZ169, and a 16-fold shift for OPC-167832. These shifts indicate a loss of 50% to 94% free drug activity in these static MIC assessments (51), which is somewhat lower than the cited values of>99% for OPC, BTZ, or PBTZ and 79% for TBA-7371, as reported by the various compound suppliers (21). This could indicate interaction with other components of human plasma that are not present in our *in vitro* serum shift assay.

BTZ-043 was found to exhibit significant efficacy in the lungs and spleens of *Mtb* Erdman-infected C3HeB/FeJ mice. With 1 month of treatment, all dose groups of BTZ-043 promoted statistically significant, albeit not dose proportional, reductions in bacterial lung loads. After 2 months of treatment, all BTZ-043 groups showed dose-proportional, highly significant reductions in lung burdens, with a maximal effect for the 200 mg/kg high-dose BTZ-043 study group (see Fig. 2). This is consistent with a previous report of the maximal effective dose of MED_99_ in mouse models without necrotic lesions (10). The calculated kill rates for the 2 months treatment study arm proved much higher and also dose-proportionate compared to the 1 month study arm. This indicates that higher BTZ-043 dose levels are associated with a faster rate of kill during the second month of treatment. A more rapid kill during month 2 of treatment was reported in a previous study for a second suicide DprE1 inhibitor, PBTZ169, which was not similarly observed for the non-covalent DprE1 inhibitors OPC-167832 and TBA-7371 in C3HeB/FeJ mice (21). It is therefore tempting to speculate that this may be a general attribute of covalent DprE1 inhibitors in C3HeB/FeJ mice. Alternate explanations, including differences in plasma protein binding or pharmacokinetic loading of tissues, cannot be ruled out.

Caseous necrotic Type I granulomas are found in the lungs only and are highly organized with a central necrotic core composed of primarily neutrophilic debris surrounded by a cellular layer, which is bordered by a collagen rim with interstitial macrophages admixed within the rim (24, 37). The cellular layer includes a foamy macrophage cell cuff immediately adjacent the collagen rim (defined here as rim), with a layer of live as well as dead, but intact, neutrophils (defined here as the cellular-caseum interface) surrounding the necrotic inner caseum consisting of acellular debris (defined here as caseum) (24) (see Fig. 4). This results in not only high numbers of extracellular Mtb in the caseum but also a large intracellular population of Mtb in the foamy macrophage and neutrophil layers. By contrast, Type III lesions found throughout the lung harbor only low numbers of Mtb, which are found to be exclusively intracellular. For some drugs, these distinct microenvironments can lead to a differential treatment response, which is not unique to the C3HeB/FeJ mouse model but has also been observed in animal models with similar pulmonary pathologies, such as the non-human primate, marmoset, and rabbit models [reviewed in reference (28)]. For instance, Mtb in rabbit caseum is largely non-replicating and exhibits extreme drug tolerance for many drugs (43). The presence of heterogenous lung pathology often impacts drug efficacy in C3HeB/FeJ mice, resulting in a bimodal treatment response in which two distinct populations of mice emerge, defined as responsive in mice lacking large necrotic lesions or less responsive in mice with caseous necrotic lesions. This is not similarly seen in spleens, which present with homogeneous, non-necrotic, cellular lesions (37). Treatment of C3HeB/FeJ mice for 2 months with BTZ-043 did not reveal clear separation between mouse populations that presented with caseous necrotic lesions versus those with less severe pathology, whereas after only 1 month of treatment, mice with caseous necrotic lesions tended to have higher overall CFU burdens, and some bimodal response could be observed (see Fig. 2). These data suggest a delayed effect of BTZ-043 killing on populations of Mtb in mice featuring larger necrotic lesions compared to those lacking visible necrotic lesions.

BTZ-043 was found to distribute well throughout the lungs and into caseous necrotic lesions. At a dose level of 100 mg/kg administered 5 out of 7 days per week for 2.5 weeks, BTZ-043 levels were highest in plasma, lung parenchyma, cellular lesion, and the cellular-caseum interface, achieving levels that were 237-, 228-, 121-, and 93-fold over the serum-shifted MIC at the T_max_ relative to peak plasma concentration, respectively. Drug levels fell below the serum-shifted MIC in each of these sampled compartments at the end of the dosing interval but were retained at levels 1.6- to 6-fold above the serum shift MIC in caseum (see Fig. 1 and 4). Given the observed lack of activity for BTZ-043 and other cell wall active agents against non-replicating Mtb in explanted rabbit caseum (Fig. 1C) (43), we hypothesize that BTZ-043 retention in non-vascularized, necrotic caseum may serve to seed surrounding foamy macrophage layers, which are highly permissive to Mtb replication (21, 45).

Histopathology analysis using two complementary approaches (i.e., semi-quantitative evaluation by a board-certified pathologist and quantitative analysis by new software called LIRA) confirmed that BTZ-043 treatment was able to halt the progression of pulmonary pathology, primarily by preventing the development of destructive Type II neutrophilic lesions. In addition, a lower incidence of Type III cellular lesions in BTZ-043-treated mice was observed compared to the start of treatment as well as the 8 weeks untreated control group, which could suggest some overall healing of lung pathology after 2 months of treatment with BTZ-043 at 100 mg/kg. It is equally noteworthy that these methods did not detect any appreciable healing or improvement of Type I disease relative to the untreated controls at the 8-week time point, which was not an unexpected result after short-term treatments using only single agents compared to more effective multidrug regimens. These data should be interpreted with caution, given the small number of animals allocated to this study arm.

Mtb-infected C3HeB/FeJ mice appear to harbor a diverse array of bacterial phenotypes, including actively replicating bacilli in the peripheral foamy macrophage layer and a population of viable extracellular bacilli in the core of mature necrotic lesions that have shown in our earlier described studies heterogeneity and dramatically slowed replication (21, 45). In the present study, BTZ-043 did not show activity against bacterial phenotypes harbored in explanted rabbit caseum (see Fig. 1) (43, 52). Given their established mechanism of action, we speculate that BTZ-043 and the three other potent DprE1 inhibitors in development (21) primarily target replicating bacteria found in immature-developing lesions *in vivo* (53) and in the cellular-caseum interface (21, 45) rather than the non-replicating bacterial subpopulations present in fully developed necrotic, hypoxic, and caseous pulmonary lesions. More studies are needed to test this hypothesis. It will also be informative to test BTZ-043 in combination with a compound or compounds that can kill non-replicating bacilli to show that cell wall inhibitors should be pursued for treatment shortening. To this end, BTZ-043 was previously reported to be fully compatible with other TB drug candidates (54), and the close analog, PBTZ169, showed favorable interactions with bedaquiline and pyrazinamide *in vivo* (15).

Similar to our results, a recent manuscript evaluated BTZ-043 for *in vivo* activity and lesion penetration in the guinea pig model for TB following surgical excision of bacillus Calmette-Guérin (BCG)-induced granulomas or granulomas arising from virulent infection, as well as draining lymph nodes and splenic tissues (48). Despite differences in maturity of granulomas at the start of treatment [2 to 2.5 months herein versus 14 to 28 days in reference (48)], both studies were highly complementary in terms of demonstrating BTZ-043 penetration into granulomatous lesions and contributions of BTZ-043 treatments to CFU reductions in different tissue microenvironments. One benefit of the LCM approach employed in our study is the ability to obtain absolute drug levels in defined areas of Type I lung lesions such as the caseum (the core of the caseous necrotic lesion), foamy macrophage rim, necrotic neutrophil rim, and uninvolved lung parenchyma without cross-contamination arising from surgical excision, which lacks such precision. Additionally, lesion distribution was sampled at the plasma PK trough and peak, allowing more precise quantitation of spatial drug distribution and assessment of drug retention in tissues over time. One limitation of both studies is an inability to discern whether the antibacterial activity of BTZ-043 in lesions is impacting all lesion-bound Mtb populations equally, but to a lesser extent than observed for splenic control tissues, or if BTZ-043 shows preferential activity for specific Mtb populations in lesions, as we have suggested here.

Overall, our report has several limitations. C3HeB/FeJ mice develop heterologous pulmonary pathology that varies between subjects within a given study group. Larger group sizes improve overall statistical power, but not all study groups show equivalent numbers of mice showing larger necrotic lesions, which impacts overall CFU burden, drug distribution into lesions, and drug activity. Drawing conclusions across such study groups is improved by retrospective classification of high and low responders using blinded gross pathology images, but this is limited only to mice showing visible necrotic lesions in digital images. Additionally, the rate of drug distribution, retention, and clearance cannot be concluded based on lesion PK studies at a single time point, as performed herein. However, these initial studies provide a basis on which to conclude that BTZ-043 is capable of penetrating cellular and necrotic lesions in C3HeB/FeJ mice.

In conclusion, the studies presented here have confirmed the good efficacy of BTZ-043 in the C3HeB/FeJ mouse model, which provides an additional preclinical data point and provides evidence that the series may prove impactful as part of a new TB regimen to treat human patients with heterogeneous lung disease.

## MATERIALS AND METHODS

### Bacterial strain

*Mtb* strain Erdman (TMCC 107) was used *in vitro* and to infect mice.

### *In vitro* activity testing

The MIC was determined for TBA-7371, OPC-167832, PBTZ169, moxifloxacin, and linezolid against *Mtb* Erdman in 7H9 media supplemented with 0.2% (vol/vol) glycerol and 10% (vol/vol) ADC, with 0.05% (vol/vol) Tween-80 (7H9 media). MICs were determined by a broth microdilution assay using twofold serial drug dilutions with an Alamar Blue endpoint (55, 56). The lowest consecutive antimicrobial concentration that did not produce a visible color change with Alamar Blue and/or showed a ≥80% reduction in OD_600_ relative to drug-free control wells was regarded as the MIC. In parallel, the MICs were also determined in the presence of 4% (wt/vol) huSA (Sigma # A1653) to assess potential protein binding (serum-shift assay). Generally, a shift in MIC of two wells (fourfold shift in MIC) is considered a significant shift due to protein binding.

The sqMBC was determined on 7H11 Nunc Omni Tray (Thermo Scientific, Waltham, MA, USA) plates supplemented with 0.2% (vol/vol) glycerol, 10% (vol/vol) OADC supplement (GIBCO BRL, Gaithersburg, MD, USA), 0.01 mg/mL cycloheximide, and 0.05 mg/mL carbenicillin with 0.4% (wt/vol) activated charcoal (Charcoal Omni) following assessment of the broth microdilution MIC on day 10 of incubation. Briefly, all assay wells are thoroughly mixed to suspend any bacterial pellets. Robot-assisted transfer and agar-touch spot methods were used to transfer 0.005 mL of each suspended well to a recipient Charcoal Omni plate. Cohesive drops are allowed to dry prior to incubation at 37°C for at least 21 days in sealed zip-top bags. The sqMBC was defined as the lowest consecutive drug concentration that resulted in a 99% (2−log_10_) CFU reduction of the starting inoculum. Moxifloxacin and linezolid were included as positive and negative MBC controls, respectively. sqMBC results were confirmed independently using conventional MBC plating methods (data not shown).

The kill kinetics of BTZ-043 against *Mtb* Erdman were determined under replicating conditions at 37°C in 7H9 media. BTZ-043 was added to actively growing cultures (~1 × 10^5^ CFU/mL) at 8× or 20× MIC. An equal volume of dimethyl sulfoxide (DMSO) (i.e., 1%) was included as a vehicle control for comparison. The number of surviving CFU/mL was determined over 14 days by serial dilution and culture on Middlebrook 7H11 agar plates supplemented with 0.2% (vol/vol) glycerol, 10% (vol/vol) OADC supplement (GIBCO BRL), 0.01 mg/mL cycloheximide, and 0.05 mg/mL carbenicillin (defined here as 7H11 agar plates), which was further supplemented with 0.4% (wt/vol) activated charcoal to prevent drug carry-over as described previously (57).

The rabbit caseum MBC assay was performed to assess the activity of BTZ-043 against non-replicating bacteria in rabbit caseum, as described previously (43). Briefly, each drug stock solution (50 mM) was serially diluted in the corresponding vehicle, and 1 µL of each dilution was dispensed in each well of a 96-well assay plate. BTZ-043 was evaluated at the final concentration range of 0.125 to 512 µM in fourfold increasing test concentrations. Frozen caseum extracted from rabbit lungs was thawed and homogenized in 2 vol of sterile water using a Fastprep-24 instrument (MP Biomedicals) and 1.4-mm-diameter ceramic (zirconium oxide) beads. Fifty microliters of caseum homogenate was dispensed in each well. The 96-well plates were incubated at 37°C for 7 days. After incubation, caseum homogenate from each well was serially diluted and plated on 7H11 agar plates in triplicate. Agar plates were incubated for up to 4 weeks before CFU counts were performed. The caseum MBC_90_ was defined as the concentration of drug required to kill 90% of *M. tuberculosis* bacteria contained in the caseum sample. The statistical significance of the differences in CFU counts between day 0 and day 7 (DMSO-only controls) was analyzed using paired *t*-tests. All procedures were performed with approval from the Hackensack Meridian Health Institutional Biosafety Committee.

### Animals

Female-specific pathogen-free C3HeB/FeJ mice, age 8–10 weeks, were purchased from Jackson Laboratories (Bar Harbor, ME). Mice were housed in an animal bio-safety level III (ABSL-3) facility employing autoclaved bedding, water, and mouse chow *ad libitum*. A specific pathogen-free status was verified by testing sentinel mice housed within the colony. All animal studies were performed at Colorado State University in a certified ABSL-3 facility in strict accordance with the regulations and recommendations of the Guide for the Care and Use of Laboratory Animals of the National Institutes of Health and the Centers of Disease Control. All procedures and protocols for infecting mice with *Mtb* and subsequent drug treatments in the described mouse infection studies were approved by the Colorado State University Institutional Animal Care and Use Committee (IACUC) (reference number of approved protocol: KP 1429). All experiments were approved by IACUC prior to the initiation of mouse studies and conducted following the relevant guidelines and regulations (reference number 1429). Mice were euthanized by CO_2_ inhalation, a method approved by the CSU IACUC.

### Aerosol infection

Mice were infected by aerosol with the *Mtb* Erdman strain (TMCC 107), and the infective bacterial inocula were prepared as described earlier (58). Briefly, bacteria were initially grown as pellicle to generate low-passage seed lots of virulent bacteria. Working stocks were generated by growing a proportion of the seed lot to mid-log phase in Proskauer-Beck medium containing 0.05% Tween 80 (Sigma Chemical Co., St. Louis, MO, USA) using three passages, enumerated by colony enumeration on 7H11 agar plates, divided into 1.5 mL aliquots, and stored at −70°C until use. C3HeB/FeJ mice were exposed to a low-dose aerosol infection using a Glas-Col inhalation exposure system, as previously described (59), resulting in an average of 60 bacteria in the lungs per mouse 1 day following aerosol exposure. Infected mice were observed and weighed at least once a week. Starting at day 21 until the start of therapy, mice were observed and weighed two to three times per week due to the increased incidence of morbidity and mortality associated with clinical TB disease. Any mice exhibiting clinical symptoms of illness were humanely euthanized. For every treatment group, eight mice per group were used to achieve sufficient statistical power in this mouse model.

### Drug efficacy experiments and bacterial enumeration

For drug efficacy experiments, C3HeB/FeJ mice from different aerosol runs were randomized (*n* = 8 per group per time point) into dosing groups. Treatments with BTZ-043 (at 50, 100, and 200 mg/kg, QD) started 8 weeks post aerosol and occurred 5 out of 7 days per week (Monday through Friday) for a total of 8 weeks. BTZ-043 was supplied formulated in 1% carboxymethylcellulose and 0.5% Tween 80. The BTZ-043 suspension was stored at 4°C, acclimated to ambient room temperature, and mixed by vortex to achieve a homogenous suspension prior to oral administration.

At the time of sacrifice, whole lungs from eight mice per treatment group were aseptically removed and disrupted with a tissue homogenizer (Precellys, Bertin Instruments, Rockville, MD, USA). Additional four mice from the high-dose group were sacrificed after 8 weeks, and their lungs were fixed for histopathology purposes (see below). To assess efficacy, the number of viable organisms was determined by plating serial dilutions of whole lungs. Due to the long half-life and high protein binding capacity of BTZ-043, lungs and spleens from all drug-treated animals as well as untreated controls were homogenized in phosphate-buffered saline (PBS) plus 10% bovine serum albumin and plated on 7H11 agar, further supplemented with 0.4% activated charcoal to prevent drug carry-over as described previously (57). Colonies were enumerated after at least 28 days of incubation at 37°C, and plates were incubated for 8 weeks to ensure that all viable colonies were detected.

### Statistical analysis

The viable CFU values were transformed to logarithms as log_10_ CFU per organ, which were evaluated by a one-way analysis of variance with adjustments for multiple comparisons using one-way Tukey’s test (pairwise comparison between all treatment groups) or Dunnett’s test (for comparison of each treatment to the untreated controls) using Prism 8 (GraphPad Software, San Diego, CA, USA). Differences were considered significant at the 95% level of confidence.

### Histological analysis

A separate group of four mice at the 200 mg/kg BTZ-043 dose level were sacrificed after 8 weeks, and their whole lungs were fixed for histopathology purposes. Four mice from the start of treatment and three untreated mice from the 8-week time point were included as controls. Lung perfusions were performed by clamping the caudal vena cava with straight-tipped hemostats to prevent 4% paraformaldehyde (PFA) from escaping into the abdominal cavity and by clamping the cranial vena cava with a clamp to retain PFA in the lungs. A scalpel was used to make a small incision into the left ventricle of the heart for blood to drain, and a syringe with a 26-gauge 1/2-inch needle was used to slowly inject 10 mL of 4% paraformaldehyde into the right ventricle for lung fixation. Lungs were then placed into a designated histology cassette in a jar of 4% PFA to fix for 48 h. After 48 h, lungs were placed into fresh 1× PBS. Whole lungs were sectioned and stained with H&E. Digital image scans from microscopic slides containing the C3HeB/FeJ lungs were generated at Premier Laboratories (Longmont, CO, USA) on an Aperio Scanscope XT digital slide scanner (Nikon, Melville, NY, USA), at 20× magnification, with an image resolution of 0.5 μm/pixel.

Histopathology analysis was performed using two different complementary approaches, as described previously (21). The first classical pathology analysis was performed on blinded samples by a board-certified pathologist specializing in *Mtb* pathology examination in mouse and guinea pig models (Dr. Brendan Podell, DMIP, CSU), using semi-quantitative scoring of lung involvement per lesion type as well as a qualitative analysis describing the specific pathology features for every slide. The second histopathology method used a newly developed pathologist-assistive software called LIRA (22), which reduces user limitations in terms of time and reproducibility while providing a rapid quantitative scoring system for digital histopathology image analysis. Both methods were performed as described previously (21).

### Collection of samples for pharmacokinetic analysis

For the PK experiments, C3HeB/FeJ mice (*n* = 8 per treatment group per time point) were dosed starting 10 wk post aerosol with BTZ-043 at 100 mg/kg, QD. Treatment occurred 5 out of 7 days per week (Monday through Friday) for a total of 2.5 week to reach steady-state drug levels. Drugs were prepared as described for the efficacy studies above. Mice were euthanized, and plasma and tissues were collected at two time points, selected based on the plasma C_max_ (1 h) and C_min_ (24 h) time points reported to us from past PK studies by the supplier.

Whole blood was obtained via cardiac puncture and processed in plasma separator tubes (Becton, Dickinson and Co., Franklin Lakes, NJ, USA), centrifuged at 3,750×RCF for 2 min at 4°C, aliquoted into Eppendorf microcentrifuge tubes, and stored at −80°C until analysis. Mice with pronounced lung pathology were selected to collect samples for spatial drug quantitation by gravity-assisted LCM. Briefly, whole lung samples consisting of the cranial, medial, and accessory lung lobes were collected on clear disposable base molds with the desired cutting surface in direct contact with the base of the tray (Fisher Scientific, Hampton, NH, USA). Collection trays are then placed onto a pre-chilled, 4-inch aluminum block in 2 inches of liquid nitrogen in a Styrofoam cooler and allowed to sit covered for 10 min. Tissue trays containing frozen lobes were wrapped in foil squares, placed individually into labeled zip-lock bags, and immediately transferred onto dry ice. Samples were stored at −80°C until analysis.

### Drug quantitation in plasma and infected tissues by HPLC coupled to tandem mass spectrometry

Drug levels in plasma were quantified by high-pressure LC-MS/MS. Drug levels in tissues were measured by spatial quantitation in thin tissue sections by LCM, followed by LC-MS/MS analysis of microdissected areas (49).

A neat 1 mg/mL DMSO stock of BTZ-043 was serially diluted in methanol (MeOH) to create standard curves and quality control (QC) solutions. Drug-free CD-1 mouse K_2_-EDTA plasma and lungs from BioIVT were used to build standard curves. Mouse lung and drug-free control mouse lung samples were weighed and homogenized in 4 vol of PBS. Homogenization was achieved using a FastPrep-24 instrument (MP Biomedicals) and 1.4 mm zirconium oxide beads (Bertin Corp.). Ten microliters of neat spiking solutions was added to 90 µL of drug-free plasma or control tissue homogenate to create standards and QC. Ten microliters of sample, standards, QC, and controls; 10 µL of 200 ng/mL BTZ-043-d4 in MeOH; and 10 µL of 250 mg/mL ascorbic acid in MQW were added to 96-well plate wells and centrifuged for 1 min at 4,000 rpm. Ascorbic acid was added to prevent conversion of the BTZ-043-M2 metabolite to BTZ-043. Eight hundred microliters of MeOH was added to each well and vortexed for 5 min. The plate was centrifuged for 5 min at 4,000 rpm, and 100 µL of supernatant was transferred into a new 96-well plate for LC-MS/MS analysis.

LC-MS/MS analysis was performed on a Sciex Applied Biosystems Qtrap 6500+ triple-quadrupole mass spectrometer coupled to a Shimadzu Nexera X2 HPLC. Chromatography was performed with an Agilent Zorbax SB-C8 column (2.1 × 30 mm; particle size, 3.5 µm) using a reverse phase gradient elution. Gradients used 10 mM ammonium acetate in Milli-Q deionized water for the aqueous mobile phase and acetonitrile for the organic mobile phase. Multiple-reaction monitoring (MRM) of parent/daughter transitions in electrospray positive-ionization mode was used to quantify the analytes. MRM transitions of 432.2/291.8 and 436.2/294.0 were used for BTZ-043 and BTZ-043-d4, respectively. Sample analysis was accepted if the concentrations of the quality control samples were within 20% of the nominal concentration. Data processing was performed using Analyst software (version 1.6.2; Applied Biosystems Sciex).

### Laser capture microdissection

Twenty-five-micrometer-thick tissue sections were cut from C3HeB/FeJ mouse lung biopsies using a Leica CM 1860UV cryostat (Buffalo Grove, IL, USA) and thawed and mounted onto 1.4-µm-thick Leica PET-Membrane FrameSlides (Buffalo Grove, IL, USA) for LCM. Tissue sections were immediately stored in sealed containers at −80°C. Adjacent 10-µm-thick tissue sections were thawed and mounted onto standard glass microscopy slides for H&E staining.

Cellular, necrotic (caseum), and uninvolved lung lesion areas totaling 3 million µm^2^ were dissected from 1 to 3 serial lung biopsy tissue sections using a Leica LMD6 system (Buffalo Grove, IL, USA) set-up within a Biosafety Level 3 facility. Areas of cellular and caseous lesions were identified optically from the brightfield image scan and by comparison to the adjacent H&E reference tissue. Pooled dissected lesion tissues were collected into 0.25 mL standard PCR tubes and immediately transferred to −80°C. Two microliters of PBS, 2 µL of 250 mg/mL ascorbic acid, 10 µL of MeOH, and 50 µL of extraction solution (MeOH) with 200 ng/mL BTZ-043-d4 and 1 ng/mL verapamil were added to each tube, which were vortexed for 5 min and centrifuged at 4,000 rpm for 5 min at room temperature. Fifty microliters of supernatant was transferred to a 96-well plate for LC/MS-MS analysis.

BTZ-043 stock and quality control solutions were prepared as described above. Ten microliters of neat spiking solutions, 2 µL of lesion homogenate, and 2 µL of 250 mg/mL ascorbic acid were added, and extraction was performed by adding 50 µL of extraction solution MeOH with 200 ng/mL BTZ-043-d4 and 1 ng/mL verapamil. Extracts were vortexed for 5 min and centrifuged at 4,000 rpm for 5 min. Fifty microliters of supernatant was transferred for LC/MS-MS analysis as described above. The total tissue volume of each pooled sample was determined based on the surface area of the pooled sections and the 25-µm tissue thickness. A dilution factor was used to normalize the tissue volumes with the standard curve for quantification.

### Calculations for the rate of killing in the lungs and spleens of C3HeB/FeJ mice

The drug kill rates for the observed lung and spleen CFU counts were calculated as the slopes of linear regression lines on the individual log_10_ CFU values using


$$\log_{10}[CFU(t)]=-k\cdot t+\log_{10}[CFU(0)],$$

where *t* ≥ 0 is the time since the start of treatment, *k* is the kill rate constant, and CFU(0) is the pre-treatment value. The kill rates represent the daily rate of decrease in log_10_ CFU (with negative values representing an increase or growth) and were calculated for the separate intervals: day 0 to day 28 (*k*_0−28_), day 28 to day 56 (*k*_28−56_), and day 0 to day 56 (*k*_0−56_), where the latter included all values for days 0, 28, and 56, respectively. The kill rates for BTZ-043 in C3HeB/FeJ mice are shown in Table S2A and S2C. Table SB shows the drug kill rates for Type I and Type III subsets identified for lung tissues.

## SUPPLEMENTAL MATERIAL

Tables S1 to S3 aac.00597-23-s0001.docx Supplemental data.

ASM does not own the copyrights to Supplemental Material that may be linked to, or accessed through, an article. The authors have granted ASM a non-exclusive, world-wide license to publish the Supplemental Material files. Please contact the corresponding author directly for reuse.
