# Supplementary material for: Drug distribution and efficacy of the DprE1 inhibitor BTZ-043 in the C3HeB/FeJ mouse tuberculosis model
Source: Antimicrob Agents Chemother. 2023 Oct 4;67(11):e00597-23. doi: 10.1128/aac.00597-23 (PMC10648937; doi:10.1128/aac.00597-23)
Supplement: Tables S1 to S3 — Supplemental data. [file aac.00597-23-s0001.docx]

**Supplemental data**

**Title:**

Drug distribution and Efficacy of the DprE1 inhibitor BTZ-043 in the C3HeB/FeJ Mouse Tuberculosis Model

**Authors:**

Michelle E. Ramey^a^, Firat Kaya^b^, Allison A. Bauman^a^, Lisa M. Massoudi^a^, Jansy P. Sarathy^b^, Matthew D. Zimmerman^b^, Dashick W. L. Scott^a^, Alyx M. Job^a^, Jake A. Miller-Dawson^a^, Brendan K. Podell^a^, Michael A. Lyons^a^, Véronique Dartois^b^, Anne J. Lenaerts^a^ and Gregory T. Robertson^a,c^

**Affiliations:**

^a^Mycobacteria Research Laboratories, Department of Microbiology, Immunology and Pathology, Colorado State University, Fort Collins, Colorado, United States of America

^b^Center for Discovery and Innovation, Hackensack Meridian Health, Nutley, New Jersey, United States of America

^c^Corresponding Author: Gregory.Robertson@colostate.edu

Keywords: Tuberculosis, Murine Models, DprE1 inhibitor, BTZ-043, C3HeB/FeJ

Running Title: DprE1 inhibitor BTZ-043 in C3HeB/FeJ mice

**Suppl. Table 1:** *P* values using a one-way ANOVA. (**A**) *P* values for 4 weeks of treatment in lungs, (**B**) *P* values for 4 weeks of treatment in spleens, (**C**) *P* values for 8 weeks of treatment in lungs, (**D**) *P* values for 8 weeks of treatment in spleens.

**A**

| Treatment/Dose (frequency) 4 weeks lungs | | Start of treatment | Untreated | BTZ-043 | | |
| --- | --- | --- | --- | --- | --- | --- |
|  |  |  |  | 50 (BID) | 100 (BID) | 200 (BID) |
| Start of treatment | |  |  |  |  |  |
| Untreated | | 0.0698 |  |  |  |  |
| BTZ-043 | 50 (BID) | 0.4382 | 0.002 |  |  |  |
|  | 100 (BID) | 0.9892 | 0.0307 | 0.8550 |  |  |
|  | 200 (BID) | 0.9928 | 0.0335 | 0.6445 | >0.9999 |  |

**B**

| Treatment/Dose (frequency) 4 weeks spleens | | Start of treatment | Untreated | BTZ-043 | | |
| --- | --- | --- | --- | --- | --- | --- |
|  |  |  |  | 50 (BID) | 100 (BID) | 200 (BID) |
| Start of treatment | |  |  |  |  |  |
| Untreated | | 0.0235 |  |  |  |  |
| BTZ-043 | 50 (BID) | <0.0001 | 0.0015 |  |  |  |
|  | 100 (BID) | <0.0001 | 0.0032 | 0.9862 |  |  |
|  | 200 (BID) | <0.0001 | 0.0001 | 0.5579 | 0.3492 |  |

**C**

| Treatment/Dose (frequency) 8 weeks lungs | | Start of treatment | Untreated | BTZ-043 | | |
| --- | --- | --- | --- | --- | --- | --- |
|  |  |  |  | 50 (BID) | 100 (BID) | 200 (BID) |
| Start of treatment | |  |  |  |  |  |
| Untreated | | 0.0051 |  |  |  |  |
| BTZ-043 | 50 (BID) | 0.0463 | <0.0001 |  |  |  |
|  | 100 (BID) | 0.0005 | <0.0001 | 0.2495 |  |  |
|  | 200 (BID) | <0.0001 | <0.0001 | 0.0326 | 0.3947 |  |

**D**

| Treatment/Dose (frequency) 8 weeks spleens | | Start of treatment | Untreated | BTZ-043 | | |
| --- | --- | --- | --- | --- | --- | --- |
|  |  |  |  | 50 (BID) | 100 (BID) | 200 (BID) |
| Start of treatment | |  |  |  |  |  |
| Untreated | | <0.0001 |  |  |  |  |
| BTZ-043 | 50 (BID) | <0.0001 | <0.0001 |  |  |  |
|  | 100 (BID) | <0.0001 | <0.0001 | 0.0101 |  |  |
|  | 200 (BID) | <0.0001 | <0.0001 | 0.0058 | 0.5142 |  |

**Suppl. Table 2:** Kill rates for BTZ-043 in C3HeB/FeJ mice after 4 and 8 weeks of treatment at 50, 100, 200 mg/kg, QD. Data represent the rate of killing and the standard error for the least squares fit to the data (SE) per day for the treatment periods [day x to day y], in log_10_CFU per day. (**A**) Drug kill rates per day in lungs, (**B**) Drug kill rates per day in Type I (T1) and Type III (TIII) lesions in lungs, (**C**) Drug kill rates per day in spleens.

**A**

| Drug (mg/kg) | k_[0-28] | k_[28-56] | k_[0-56] |
| --- | --- | --- | --- |
| PreRx & Untreated | -0.038 (0.014) | -0.011 (0.015) | -0.025 (0.007) |
|  |  |  |  |
| BTZ-043 (50) | 0.022 (0.018) | 0.015 (0.021) | 0.019 (0.009) |
| BTZ-043 (100) | 0.006 (0.013) | 0.056 (0.015) | 0.031 (0.007) |
| BTZ-043 (200) | 0.005 (0.010) | 0.087 (0.013) | 0.046 (0.008) |

**B**

| Drug (dose mg/kg) |  | k_[0-28] | k_[28-56] | k_[0-56] |
| --- | --- | --- | --- | --- |
| PreRx & Untreated | TI | -0.041 (0.016) | 0.011 (0.012) | -0.013 (0.008) |
|  | TIII | -0.027 (0.012) | -0.032 (0.025) | -0.029 (0.008) |
|  |  |  |  |  |
| BTZ-043 (50) | TI | 0.011 (0.010) | 0.032 (0.015) | 0.023 (0.008) |
|  | TIII | 0.063 (0.007) | 0.005 (0.010) | 0.039 (0.007) |
| BTZ-043 (100) | TI | 0.011 (0.011) | 0.078 (0.017) | 0.047 (0.011) |
|  | TIII | 0.041 (0.007) | 0.003 (0.015) | 0.022 (0.005) |
| BTZ-043 (200) | TI | 0.023 (0.011) | 0.062 (0.014) | 0.042 (0.009) |
|  | TIII | 0.019 (0.009) | 0.079 (0.015) | 0.049 (0.005) |
|  |  |  |  |  |

**C**

| Drug (mg/kg) | k_[0-28] | k_[28-56] | k_[0-56] |
| --- | --- | --- | --- |
| PreRx & Untreated | -0.024 (0.011) | -0.010 (0.012) | -0.017 (0.005) |
|  |  |  |  |
| BTZ-043 (50) | 0.048 (0.006) | 0.004 (0.007) | 0.026 (0.004) |
| BTZ-043 (100) | 0.046 (0.006) | 0.036 (0.006) | 0.041 (0.003) |
| BTZ-043 (200) | 0.057 (0.005) | 0.034 (0.006) | 0.046 (0.003) |

**Suppl. Table 3:** Histopathology analysis of pulmonary sections of C3HeB/FeJ mice at the start of treatment (A) or in untreated controls and the BTZ-043 treatment group (at 200 mg/kg, QD) 8 weeks after the start of therapy (B, C, respectively). Lesion Recognition Image Analysis (LIRA) quantifies the percent lung involvement of the indicated histopathology features.

| **A** | **Group** | **percent lung involvement** | | | | |
| --- | --- | --- | --- | --- | --- | --- |
|  | Start of treatment | Type I - Caseum | Type II | Type III | Type I - Rim | Healthy Tissue |
|  | Mx I | 0.00 | 0.00 | 25.08 | 0.00 | 74.92 |
|  | Mx J | 6.31 | 0.00 | 27.77 | 3.92 | 62.00 |
|  | Mx K | 1.77 | 0.00 | 21.09 | 1.35 | 75.79 |
|  | Mx L | 19.85 | 0.00 | 15.84 | 15.32 | 48.99 |
|  | **average** | **6.98** | **0.00** | **22.45** | **5.15** | **65.43** |
|  | **SEM** | **4.49** | **0.00** | **2.59** | **3.49** | **6.32** |
| **B** |  |  |  |  |  |  |
|  | Untreated (8 weeks) | Type I - Caseum | Type II | Type III | Type I - Rim | Healthy Tissue |
|  | Mx I | 11.86 | 10.80 | 21.77 | 6.19 | 49.39 |
|  | Mx J | 1.32 | 0.00 | 38.19 | 1.11 | 59.38 |
|  | Mx K | 10.87 | 28.13 | 35.38 | 6.16 | 19.46 |
|  | **average** | **8.02** | **12.98** | **31.78** | **4.48** | **42.74** |
|  | **SEM** | **3.36** | **8.19** | **5.07** | **1.69** | **11.99** |
| **C** |  |  |  |  |  |  |
|  | BTZ-043 (200 mg/kg; 8 weeks) | Type I - Caseum | Type II | Type III | Type I - Rim | Healthy Tissue |
|  | Mx I | 2.82 | 0.00 | 2.83 | 3.75 | 90.59 |
|  | Mx J | 6.51 | 0.00 | 22.03 | 4.76 | 66.70 |
|  | Mx K | 30.69 | 0.00 | 11.21 | 10.89 | 47.22 |
|  | Mx L | 0.00 | 0.00 | 9.69 | 0.00 | 90.31 |
|  | **average** | **10.01** | **0.00** | **11.44** | **4.85** | **73.70** |
|  | **SEM** | **7.02** | **0.00** | **3.97** | **2.26** | **10.45** |
